# Binding site of restriction-modification system controller protein in Mollicutes

**DOI:** 10.1186/s12866-017-0935-4

**Published:** 2017-01-31

**Authors:** Gleb Y. Fisunov, Daria V. Evsyutina, Valentin A. Manuvera, Vadim M. Govorun

**Affiliations:** 1 Federal Research and Clinical Centre of Physical-Chemical Medicine, Malaya Pirogovskaya 1a, Moscow, 119992 Russia; 2 Department of Bioengineering and Bioinformatics, Lomonosov Moscow State University, Leninskiye Gory, GSP-1, 73, Moscow, 119234 Russia

**Keywords:** Mollicutes, Mycoplasma, Regulation, Transcription factor, Restriction-modification

## Abstract

**Background:**

Bacteria of the class Mollicutes underwent extreme reduction of genomes and gene expression control systems. Only a few regulators are known to date. In this work, we describe a novel group of transcriptional regulators that are distributed within different Mollicutes and control the expression of restriction-modification systems (RM-systems).

**Results:**

We performed cross-species search of putative regulators of RM-systems (C-proteins) and respective binding sites in Mollicutes. We identified a set of novel putative C-protein binding motifs distributed within Mollicutes. We studied the most frequent motif and respective C-protein on the model of *Mycoplasma gallisepticum S6*. We confirmed our prediction and identified key nucleotides important for C-protein binding. Further we identified novel target promoters of C-protein in *M. gallisepticum*.

**Conclusions:**

We found that C-protein of *M. gallisepticum* binds predicted conserved direct repeats of the (GTGTTAN_5_)_2_ motif. Apart from its own operon promoter, HsdC can bind to the promoters of the *clpB* chaperone gene and a tRNA cluster.

**Electronic supplementary material:**

The online version of this article (doi:10.1186/s12866-017-0935-4) contains supplementary material, which is available to authorized users.

## Background

Mollicutes are wall-less bacteria with a significantly reduced genome. The repertoire of gene expression regulators in Mollicutes is reduced as well. The smallest number of transcription factors is observed within Mycoplasmatales [1]. However, mycoplasmas are efficient parasites that colonize numerous vertebrate hosts. The ability of mycoplasmas to adapt to different conditions contrasts with the small amount of regulators. Mycoplasmas are also used as model objects for systems biology to study the core organization of living cells. Thus, the study of transcriptional regulation in mycoplasmas contributes to two topics: the adaptation of an efficient parasite using a minimal amount of regulators and the organization of gene expression regulation in the core cellular machinery.

Restriction-modification systems are widespread in bacteria. They have two enzymatic activities: site-specific DNA methylase and site-specific endonuclease. Generally, RM-systems modify hemimethylated DNA and cleave unmethylated DNA. The majority of RM-systems belong to type I or type II. While all RM-systems consist of restriction (R) and modification (M) subunits, type I systems feature an additional specificity (S) subunit as a separate protein [2, 3]. Type I RM-systems work as multimeric complexes of 2 M-1S-2R composition and recognize asymmetric sites [3]. Enzymes of type II RM-systems work separately and recognize short palindromes. Particular members of RM-systems family may exhibit different functions, including defense from exogenous DNA such as the DNA of bacteriophages [4], control of DNA replication [5] and being a selfish element [6]. RM-systems may also have specific transcriptional regulators [7, 8]. In this work, we studied the transcriptional regulators of restriction-modification systems (RM-systems) across the Mollicutes. We used *Mycoplasma gallisepticum* as a model organism to study the binding properties of the respective regulator.

A set of various RM-systems, predominantly of type II, feature specific transcriptional regulators termed controller proteins or C-proteins [7, 8], which may serve as transcriptional repressors or activators depending on the particular protein and condition [8, 9]. The binding site of C-proteins, termed the C-box, is conserved across different bacteria [10, 11]. It consists of two inverted repeats with an AGTC consensus core element. The type of C-protein binding to the C-box may govern its role as a repressor or an activator [7, 10]. The molecular mass of C-proteins is very low, and they seem to have no additional domains except a solely DNA-binding helix-turn-helix (HTH) domain. The mode of regulation depends on the protein’s synthesis and degradation speed and a feedback loop with its own promoter rather than external stimuli [8, 9]. In the RM-systems studied to date, C-protein forms an operon with the restriction subunit but not the modification subunit [10, 11]. This configuration assists the attenuation of restriction subunit concentration, which makes the methylation of genomic DNA a preferred process over its cleavage. In the current work, we used *Mycoplasma gallisepticum S6* as a model to study the DNA-binding properties of its C-protein homolog, further termed HsdC (GCW_02350) because in this bacterium it resides within the *hsd* operon (GCW_02350-GCW_02365). As a result, we identified a novel C-protein binding site.

## Methods

### Cloning and purification of HsdC protein

Cloning and purification procedures were performed as described in [12]. The HsdC (GCW_02350) coding sequence was amplified from the genomic DNA of *M. gallisepticum S6* (forward primer: ATTAGGATCCATGTTTGATTATGCAAAGAAAATTA, reverse primer: TATAGTCGACATCATCTAATTTCATGCCAATCT, sequences for cloning are underlined). The amplicon was cloned into the pETm plasmid with C-terminal His-tag as described previously [12]. HsdC protein was produced in *E. coli* BL21-Gold (DE3) cells. Cells were grown overnight, harvested by centrifugation, washed in PBS and lysed with Branson 250 Sonifier (Branson) at 22 kHz for 10 min. The lysate was diluted with sample buffer (final concentrations of 20 mM Na_2_HPO_4_, 10 mM imidazole, 500 mM NaCl, pH 7.5). The protein was purified on a Tricorn 5/50 column (GE Healthcare) with Ni Sepharose High Performance (GE Healthcare) resin using the AKTA FPLC system (GE Healthcare). After the application of lysate, the column was washed with 25-ml aliquots of sample buffer, then with wash buffer (20 mM Na_2_HPO_4_, 25 mM imidazole, 500 mM NaCl, pH 7.5) and finally with elution buffer (20 mM Na_2_HPO_4_, 500 mM imidazole, 500 mM NaCl, pH 7.5). After elution, the protein was 60-fold diluted with 20 mM Tris-HCl buffer, pH 7.5 to 20 pmol/μl and directly used for electrophoretic mobility shift assay (EMSA).

### Electrophoretic mobility shift assay

A 20-pmole aliquot of the purified protein was incubated with 10 pmole of the dsDNA oligo for 15 min at 37 °C. The binding buffer contained 20 mM Tris-HCl (pH 7.5), 100 mM KCl, and 6% glycerol. Electrophoresis was performed using the PROTEAN II xi electrophoretic cell (Bio-Rad), TB buffer (0.5 M Tris-base, 0.5 M boric acid, pH 8.3), and 6% acrylamide gel for 1 h at 450 V at 10 °C. After electrophoresis, the gel was stained with an ethidium bromide solution (3 μl of 1% EtBr per 400 ml of TB buffer) for 5 min and then visualized on a Typhoon scanner. The resulting images were analyzed using the ImageQuant software. All oligonucleotides used for EMSA are summarized in Table 1.

**Table 1:** Oligonucleotides used for EMSA experiments (only plus strand is shown)

Oligonucleotide	Sequence
P_*hsdC*_ -WT	TTATCGGCTTTGTGTTAAAATAGTGTTAACGATTTTGAAG
P_*hsdC*_ -mut1	TTATCGGCTTTCTGTTAAAATACTGTTAACGATTTTGAAG
P_*hsdC*_ -mut2	TTATCGGCTTTGTCTTAAAATAGTCTTAACGATTTTGAAG
P_*hsdC*_ -mut3	TTATCGGCTTTGTGTACAAATAGTGTACACGATTTTGAAG
P_*hsdC*_ -mut4	TTATCGGCTTTGAGTTAAAATAGAGTTAACGATTTTGAAG
P_*hsdC*_ -mut5	TTATCGGCTTTGTGATAAAATAGTGATAACGATTTTGAAG
P_*hsdC*_ -mut6	TTATCGGCTTTACCTATAAATAGTGTTAACGATTTTGAAG
P_*hsdC*_ -mut7	TTATCGGCTTTGACTTAAAATAGTGTTAACGATTTTGAAG
P_*hsdC*_ -mut8	TTATCGGCTTTGAGTTAAAATAGTGTTAACGATTTTGAAG
P_*hsdC*_ -mut9	TTATCGGCTTTGTCTTAAAATAGTGTTAACGATTTTGAAG
Neg	AAAACACCCTATTTTTGATATGATATAGTCATACAAAGGA
P_*mraZ*_	AATTCAAAAGTGTTAAAAAGTGTGAGAAAGTGGGAAAAAT
P_*clpB*_	TAATAGCCTAAGTGCTAATTTTTTGTTATAATAAATCTAT
P_*trnM*_	ATTGTTATTATATGATAATAATGTGTAACACATCGCGGGA

The HsdC binding constant was determined from the titration curve with a series of protein dilutions as described previously [12]. Briefly, the equilibrium equation was solved for the DNA-protein complex concentration to obtain the equation for the fractional saturation of DNA, which was measured in EMSA experiments (equation 2 in [12]). Then, the binding constant was determined by nonlinear regression of the experimental data to a theoretical curve by the least squares method.

### Whole-genome mapping and quantification of transcription start sites

The data on promoters’ position and activity was taken from our previous work [1]. Briefly total RNA was extracted from the cells by TrizolLS (Life Technologies) reagent, depleted with tRNAs using PureLink RNA mini spin columns (Life technologies) and fragmented by ZnSO4 treatment. Fragments were end-repaired by T4 polynucleotide kinase and treated with Terminator (Epicentre) 5′-phosphate dependent exonuclease. This procedure resulted in degradation of all but primary 5′-end fragements. Than fragments were treated by tobacco acid phosphatase (Epicentre) and ligated into adapters for RNA-seq. After cDNA synthesis and amplification cDNA libraries were subjected to normalization (removal of cDNA of rRNAs) by double-strand specific nuclease DSN (Eurogen) as described in [1]. Libraries were sequenced on SOLiD 4 platform. Sequence coverage of 5′-end enriched libraries formed sharp peaks on the positions of transcription start sites. Peaks were picked using the algorithm described in [1]. Peak coverage corresponded to the promoter activity as it was demonstrated in [1].

### Identification of HsdC homologs and RM-system components homologs in Mollicutes

Search for HsdC (C-protein) homologs was performed by NCBI blastp algorithm. Search for homologs of RM-systems proteins was performed using domain annotation from NCBI CDD database. Putative C-protein binding sites were identified by alignment of upstream regions of HsdC homologs in different species of Mollicutes.

### Data access

The raw data on transcription start sites (TSS’s) mapping was uploaded to NCBI SRA database under project id PRJNA325091.

## Results

### Distribution and conservation of C-protein homologs in Mollicutes

**Fig. 1:**
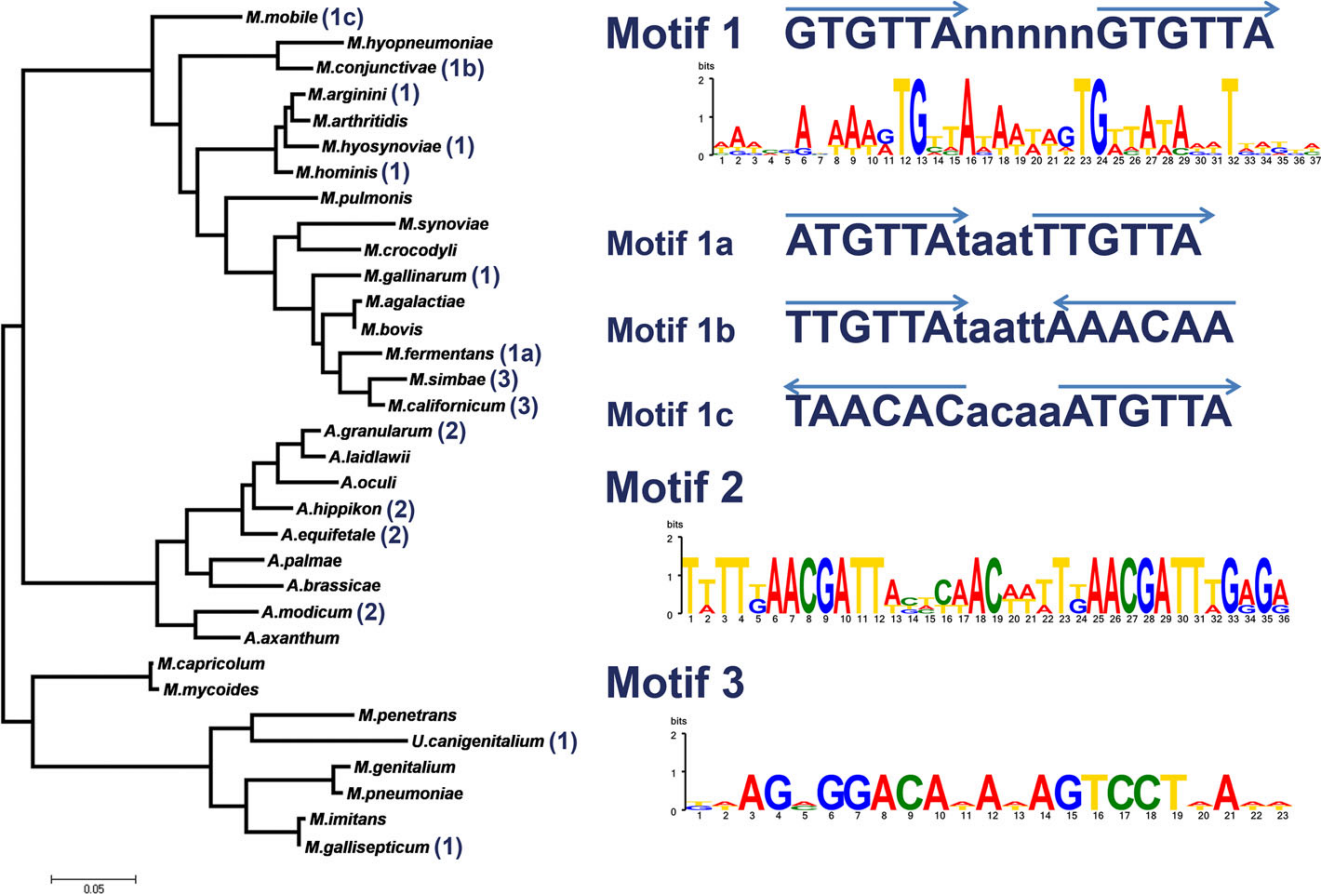
Distribution of controller protein (C-protein) homologs in Mollicutes. Phylogenetic tree was built based on 16S rRNA sequences. All displayed species have at least one RM-system (see Additional file 1: Table S1 for more detail). Presence of C-protein and its predicted binding site is indicated by number in brackets, which corresponds to a motif sequence on the right. In the current work we focused on the most frequent motif 1 (distributed within mycoplasmas). It has also several deviations: shorter spacer between repeats and inverted repeats instead of direct, but with the same consensus. There is acholeplasma-specific motif 2. Motif 3 is shared by M. symbae and M. californicum, but is completely different from the rest. However it is similar to the motif of C-protein of AdhI RM-system

Searching for HsdC homologs within Mollicutes identified a set of putative RM-system regulators. Homologs of C-proteins were distributed within different groups of Mollicutes, including Acholeplasmatales and Mycoplasmatales (Fig. 1, Table 2, Additional file 1: Table S1), but their similarity was not very high (60–40% on average). All identified proteins were associated with RM-systems, either operons or individual genes. Several HsdC homologs were found fused with modification subunits. In Acholeplasmas and several mycoplasmas, they are found within *hsd* operons (type I RM-system), while in other mycoplasmas, they are associated with type II DNA-methylases as well.

**Table 2:** Distribution of controller protein (HsdC) homologs and their predicted binding sites in Mollicutes. HsdC binding sites are underlined

Species	RM-system	Sequence
*A. equifetale*	Type I	CAATTTAACGATTTGCTACTTTTGAACGATTATTCAACAAATGAACGATTAGGGG
*A. hippikon*	Type I	TATTTAACGATTACCATACTTTTTAACGATTTGACAACAATTTAACGATTTGAGA
*A. granularum*	Type I	TTTTATTTTGTTAGTCAACTATTTAACGATTACCTTACTTTTTAACGATTTGAGA
*A. modicum*	Type I	TTTGAACGTTTAGGGCTAGTTTTGAACGATTACTCAACTTTTGAACGATTTGAGA
*M. arginini*	Type I	ATAGCAGAAAGTGCTATAATAGTGTATAATATAATCA
*M. conjunctivae*	Type I	CTCAAGTTTTTTGTTATAATTAAACAAGATAAAAAGC
*M. fermentans*	Type I	AAAAAATAAAATGTTATAATTTGTTATAAGTTGTTAG
*M. gallinarum*	Type I	CGACTAAAAAGTGCTAAAAATGTGATATAATTGTGGC
*M. gallisepticum*	Type I	TATCGGCTTTGTGTTAAAATAGTGTTAACGATTTTGA
*M. callifornicum*	Type II fusion^a^	TGTCAAGTAGAGGACATAAAGTCCTTATATCAGCT
*M. hominis*	Type II	AATCGACAAAGTGATAGTTTTGTGATATAGTTAAGAT
*M. hyosynoviae*	Type II	TATAGAGTAAATGTAATAAAAATGATATAATTTTGTC
*M. mobile*	Type II fusion^a^	TTGTATATTTTAACACACAAATGTTATAATGTAATT
*M. simbae*	Type II fusion^a^	TGTTAATAAGCGGACAAATAGTCCTAAATTAATAA
*U. canigenitalium*	Type II fusion^a^	TAGTTATAATATGTCATATTAGTGTCATATATGAATT

Controller-protein binding repeats are underlined

A. – *Acholeplasma*, M. – *Mycoplasma*, U. – *Ureaplasma*

^a^fusion of controller protein with methylase of RM-system

Cross-species analysis of the respective promoters revealed putative HsdC binding motifs (Fig. 1). To enhance the identification of promoters in Mollicutes, we used previous data on the promoter structure of *M. gallisepticum* [1]. The binding site of the well-studied C-proteins or C-box consists of at least two inverted repeats with a GACT core element, which may have extensions in some cases [10, 11]. Cross-species conservation analysis revealed several types of repeats in the promoters of operons coding for RM-systems components (Table 1). The identified motifs were not predicted in previous large-scale cross-species analysis of putative binding sites of C-proteins [13]. All studied Acholeplasmatales featured direct repeats of the (AACGAATN_12_)_3_ sequence, although the spacer length varied by 1 nt in some occasions. However, at least two repeats featured a conserved 12 nt spacer. In mycoplasmas, we observed two major variants of the motif. One variant consists of a GTGTTA core sequence forming either direct repeats (GTGTTAN_5_)_2_ or inverted repeats. The latter variant was found in *M. conjunctivae* and in *M. mobile*. Another type of motif is completely different and consists of inverted repeats, GGACN_5_GTCC. This motif is similar to the well-characterized C-box of the AhdI RM-system AGTCCN_2_GGACT [11], but with the reverse order of repeats.

### HsdC recognizes direct repeats in the promoter of hsd operon

**Fig. 2:**
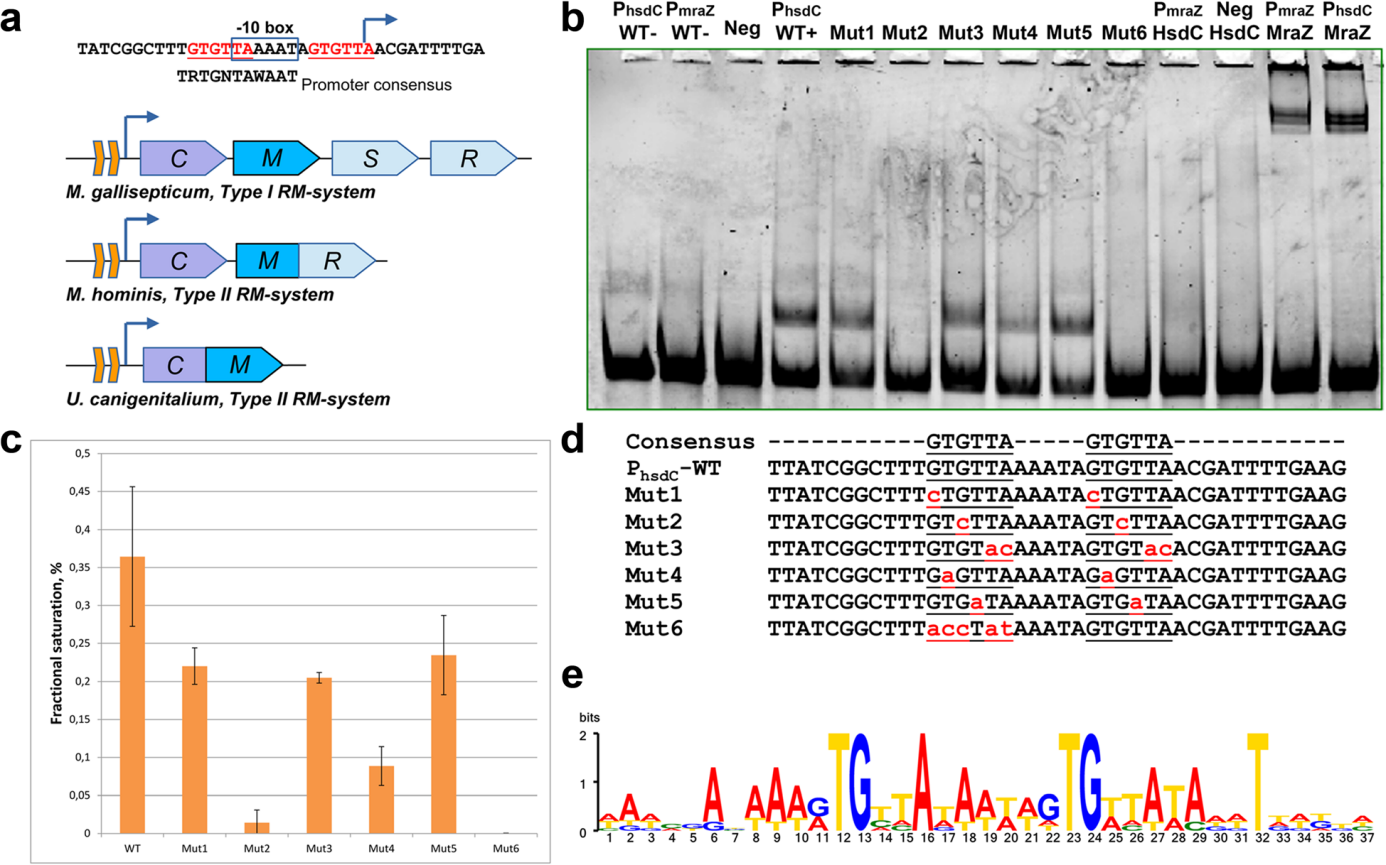
Binding site of HsdC. **a** – structure of hsd operon promoter and the diversity of operons controlled by its homologs in Mollicutes. The HsdC binding site overlaps with the -10 box and the transcription start site (promoter data were taken from [1]). Orange boxes on operons diagram show HsdC-binding repeats. **b** – EMSA of HsdC with a set of oligos including wild-type promoter of *hsd* operon, mutated variants and MraZ regulator binding site. P_hsdC_WT- – free oligo with *hsd* operon promoter sequence, P_mraZ_WT- – free oligo with DCW operon promoter sequence, Neg – control oligo without HsdC binding site, P_hsdC_WT+ – *hsd* operon promoter with HsdC protein, Mut1-6 – mutated *hsd* operon promoter with HsdC protein (sequences shown below), P_mraZ_ HsdC – DCW operon promoter with HsdC protein, Neg HsdC – control oligo with HsdC protein, P_mraZ_ MraZ – DCW cluster operon promoter with MraZ protein (as control), P_hsdC_ MraZ – *hsd* operon promoter with MraZ protein. **c** – fractional saturation of DNA of wild-type and mutated *hsd* operon promoter sequence. Sequences are shown on panel E. Data were calculated from EMSA gels. **d** – mutations introduced to *hsd* operon promoter to test HsdC specificity. **e** – sequence logo of HsdC binding site with direct repeats across the mycoplasmas. The most conserved TG dinucleotide plays the most important role in binding, as shown in A and B (Mut2 and Mut4 oligos)

In *M. gallisepticum S6,* the C-protein homolog resides within one of the *hsd* operons coding for the type I RM-system. To identify the *hsd* operon promoter, we used a previously constructed whole-genome promoter map of *M. gallisepticum S6* [1]. There are two promoters: the first is strong and lies upstream of HsdC, and the second is weak and occurs within its coding sequence. Both allow the transcription of downstream *hsdMSR* genes. The first promoter encompasses direct repeats of the (GTGTTAN_5_)_2_ sequence, which partially overlap with the core promoter (Fig. 2a). We used an electrophoretic mobility shift assay (EMSA) to study the DNA-binding properties of HsdC (Fig. 2b, c). The second and third positions within the repeat (TG dinucleotide) were the most crucial for site recognition. The mutation of either prevented HsdC binding near completely. Single mutation of G nucleotide of TG core in one repeat is sufficient to drastically impede binding (Additional file 2: Figure S1). Disruption of one of the repeats also fully prevented binding. The remaining positions within the repeat were found to be less important and they could be mutated with only moderate loss of HsdC affinity. Similar conclusions can be derived from the cross-species repeat conservation, where the central TG dinucleotide shows absolute conservation in the binding sites of motifs of similar type (Fig. 2e). We determined the binding constant of HsdC protein to the *hsd* promoter as previously described for the MraZ regulator. The binding constant was in the nanomolar range, approximately 5 × 10^−9^ M (Additional file 3: Figure S2).

To test HsdC activity *in vivo*, we used a previously described overexpression vector [12] with cloned *hsdC* ORF. However, all attempts produced a lethal phenotype. The quantitative data on *hsdC* promoter activity [1] support its role as a transcriptional repressor. The corresponding promoter features strong determinants including a consensus −10 box, extension and initiator nucleotide (consensus: TRTGNTAWAATN_6_R, *hsdC* promoter: TGTGTTAAAATN_6_A). The activity of the *hsdC* promoter (measured as coverage, see [1]) was approximately 2 orders of magnitude lower than the average activity of a promoter with the given sequence (Additional file 4: Figure S3).

### Additional targets of HsdC in M. gallisepticum genome

The HsdC binding site resembles the core binding site of the MraZ transcriptional regulator (AAAGTGKN_3_)_3_, K = G or T [12]. However, the spacer between the GTG core repeats in the motifs differs by 1 nt. We tested each protein for binding to the sites of the other (Fig. 2b, lanes P_mraZ_ HsdC – P_hsdC_ MraZ). MraZ protein is capable of binding to the HsdC motif with comparable strength as to its own (as a single octamer), while HsdC cannot bind to the MraZ motif. The MraZ-overexpressing strains obtained in our previous work [12] demonstrate no effect on the *hsd* operon *in vivo* (data not shown).

**Fig. 3:**
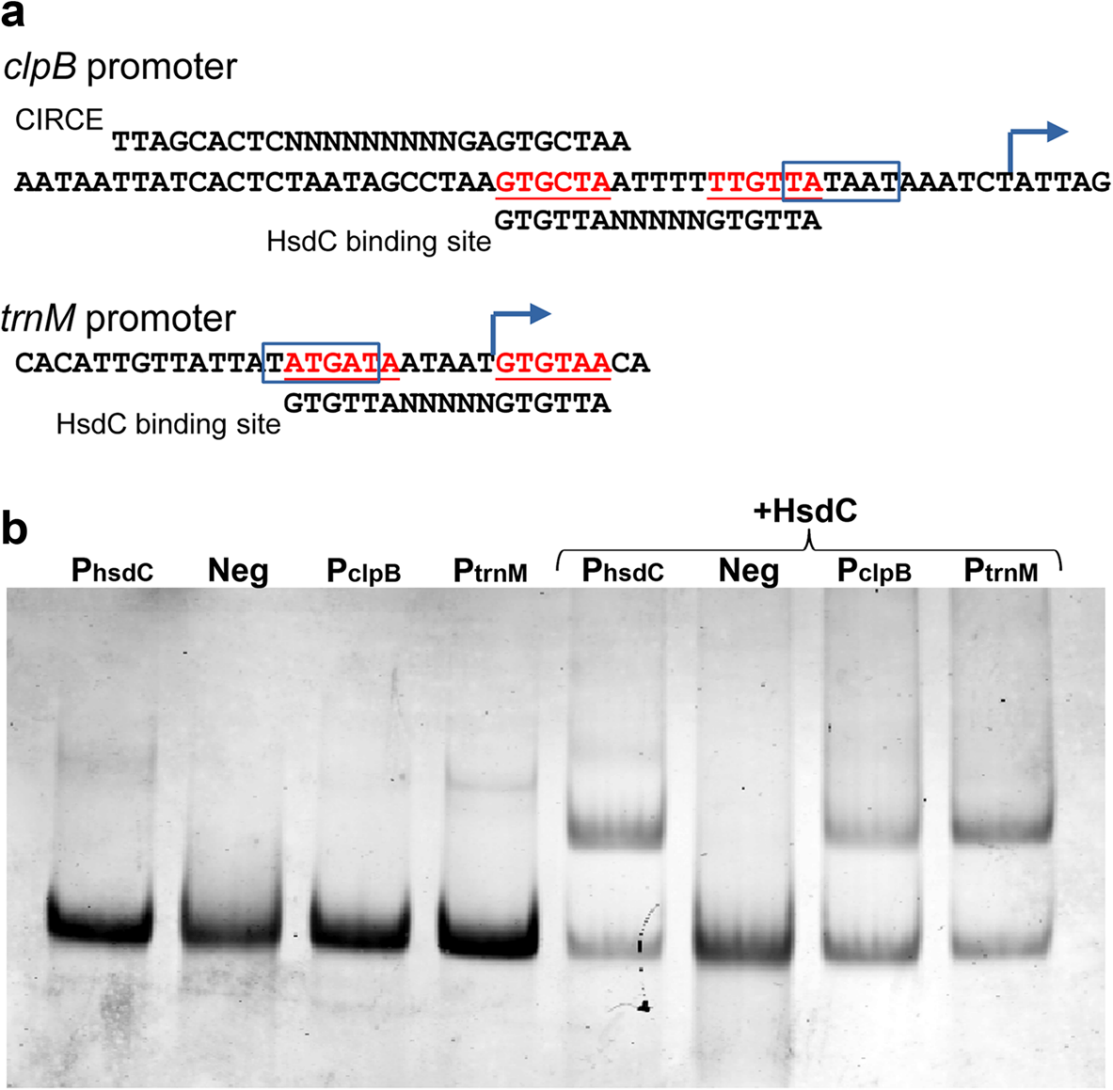
Additional binding sites of HsdC in *M. gallisepticum S6*. **a** – position of HsdC binding site in the promoters of *clpB* and *trnM* genes; *clpB* has an additional CIRCE (Controlling Inverted Repeats of Chaperone Expression) sequence, the binding site of the HrcA regulator. **b** – EMSA of HsdC with *clpB* and *trnM* promoters. PhsdC – WT promoter of hsd operon, Neg – negative control oligo, PclpB – *clpB* promoter, PtrnM – promoter of tRNA cluster (first gene *trnM*, GCW_00940)

The determination of HsdC requirements and constraints for the DNA binding allowed us to identify its additional targets in the genome of *M. gallisepticum*. The important conclusion from the binding experiments was that the central TG dinucleotide is crucial for binding, while other positions including the first G may have substitutions. We considered only promoters (identified in [1]) where the HsdC binding site resided in the vicinity of the −10 box or overlapped with transcription start site. We identified two potential targets: the tRNA operon starting with tRNA-Met (*trnM*, GCW_00940) and the *clpB* chaperone gene. In both cases, the HsdC binding site overlapped with the -10 box of the promoter. To test their functionality, we performed EMSA as described above (Fig. 3). In both cases, we detected similar or slightly weaker binding compared to the *hsd* operon promoter.

## Discussion

We identified a novel subfamily of transcriptional regulators of RM-systems, which are distributed within Mollicutes. The majority of them recognize motifs that are completely different from the known C-box in sequence and structure. There are three types of C-protein binding motifs in Mollicutes, and the most frequent ones consist of direct rather than inverted repeats. The recognition of direct rather than inverted repeats suggests an alternative protein dimerization mechanism as well. The exact role of HsdC in the control of the *hsd* operon is unclear. Extensively studied C-proteins of the AhdI [9] and PvuII [10] RM-systems serve as activators when binding upstream to the −35 region. The C-protein of the AhdI RM-system may also serve as a repressor if binds between the −35 and −10 regions, physically hampering RNA-polymerase binding. At the same time, both RM-systems consist of two transcription units, while the C-protein controls only the R-subunit gene. This configuration causes the S and M subunits to be expressed first and modify genomic DNA before the R subunit can cleave it. HsdC of *M. gallisepticum* seems to act as a repressor of the whole operon of S, M and R subunits, and no other promoters were identified in the vicinity of the HsdC binding site. It is a question if HsdC functions only as auto-repressor of the *hsd* operon or is regulated on the post-translational level. In the first case it may produce burst-like expression rather than steady one.

Restriction-methylation systems are widespread in Mollicutes, but only a few of them are controlled by a transcription factor (Additional file 1: Table S1). The *M. gallisepticum S6* strain used in this work is currently the only strain of *M. gallisepticum* that features an RM-system with a transcriptional regulator. At the same time, all strains including *S6* have another RM-system of type I that lacks a controller subunit. This observation as well as the duplication of the genomic region around the controlled RM-system indicates horizontal transfer of the whole operon (Additional file 5: Figure S4). RM-systems resemble toxin-antitoxin systems in terms of their selfish behavior [6]. However, our experiments demonstrate that HsdC can bind to promoters of important genes, including *clpB* and the tRNA gene cluster. This finding may explain the lethal phenotype of HsdC-overexpressing strains. While the functions of ClpB could be performed by other chaperones, including DnaK and the GroEL complex, only one copy each of tRNA-Asp and tRNA-Phe exist in the genome, and their function suppression cannot be compensated.

The binding site of HsdC in the *clpB* promoter overlaps with the binding site of its specific regulator HrcA, which likely leads to competition between the two proteins for the promoter. This effect at least introduces a novel mode to the regulation of *clpB*. However, even if the effect of HsdC competence is negative, it is likely that the elimination of its binding site by mutation would produce even more negative consequences. In the case of the *clpB* promoter, the core TG dinucleotides of the HsdC binding site are formed by −10 box extension and a core element of CIRCE (Controlling Inverted Repeats of Chaperone Expression), the binding site of HrcA repressor(Fig. 3a). Thus, mutation would either decrease the promoter strength or hamper HrcA-dependent regulation. Measurement of *clpB* promoter activity indicate relatively high expression level in comparison to *hsd* operon promoter (Additional file 6: Table S2). Probably it is a result of HsdC displacement by HrcA, the designated regulator of this promoter. In the case of *trnM*, the HsdC binding site could be eliminated by a mutation in first repeat, where substitution of G within the −10 box would not impede the promoter activity (Fig. 3a).

HsdC provides an example of an interesting evolutionary event: the acquisition and domestication of a foreign regulator. The promoter sequences of the *clpB* and *trnM* genes are identical in the *M. gallisepticum S6* and *R(low)* strains. This observation means that the ability to bind a novel regulator was acquired previously by chance. In the case of the *clpB* gene, the HsdC binding site was generated by an extended −10 box and a part of CIRCE, its dedicated regulator. This phenomenon may be considered as an event of exaptation [14], where adaptation to a certain type of promoter regulation leads to susceptibility to another one, which can potentially be acquired in the future.

## Conclusions

Mollicutes feature homologs of the controller protein (C-protein) of RM-systems that regulate the transcription of both type I and type II RM-systems. In some cases, they form fusions with the corresponding methylase. There are three types of C-protein binding sites: one is characteristic of Acholeplasmas, (AACGAATN_12_)_3_, and the others of mycoplasmas, GGACN_5_GTCC and (GTGTTAN_5_)_2_. The latter (the most frequent) was confirmed experimentally. The central TG dinucleotide was the most conserved and the most important for binding. Since the binding site of this type is relatively simple, the C-protein may bind other promoters, basically ones with a TRTG extension of the −10 box.

## Additional files

Additional file 1: Table S1. Distribution of restriction-modification proteins in Mollicutes. (PDF 89 kb)
Additional file 2: Figure S1. EMSA with mutations in a single repeat of HsdC binding site. (PDF 143 kb)
Additional file 3: Figure S2. Binding constant determination for HsdC. (PDF 213 kb)
Additional file 4: Figure S3. Distribution of promoter powers with strong consensus. (PDF 157 kb)
Additional file 5: Figure S4. Genomic rearrangements near *hsd* (GCW_02350 – GCW_02365) operon insertion. (PDF 176 kb)
Additional file 6
**Table S2.** Distribution of promoter strengths with strong consensus (TRTGNTAWAATN_6_R). (PDF 85 kb)
