# Supplementary material for: Binding site of restriction-modification system controller protein in Mollicutes
Source: BMC Microbiol. 2017 Jan 31;17:26. doi: 10.1186/s12866-017-0935-4 (PMC5282649; doi:10.1186/s12866-017-0935-4)
Supplement: Additional file 1: Table S1. — Distribution of restriction-modification proteins in Mollicutes. (PDF 89 kb) [file 12866_2017_935_MOESM1_ESM.pdf]

Supplementary tables

Supplementary table 1. Distribution of restriction-modification proteins in Mollicutes. If at least one protein of particular type is encoded within the genome it is marked with 1, F indicates protein fusion. Columns: C – controller subunit (transcription factor), M I – methylase of type 1 RM-system, S I – specificity subunit of type 1 RM-system, R I – restrictase subunit of type 1 RM-system, M II - methylase of type 2 RM-system; Sequence – putative controller subunit binding sites upstream of respective RM-operon.

Species	C	M I	S I	R I	M II	Sequence
A. axantum	0	0	0	0	1	
A. brassicae	0	1	1	1	1	
A. equifetale	1	1	1	1	0	CAATTTAACGATTTGCTACTTTTGAACGATTATTCAACAAATGAACGATTAGGGG
A. hippikon	1	1	1	1	0	TATTTAACGATTACCATACTTTTTAACGATTTGACAACAATTTAACGATTTGAGA
A. granularum	1	1	1	1	0	TTTTATTTTGTTAGTCAACTATTTAACGATTACCTTACTTTTTAACGATTTGAGA
A. laidlawii	0	1	1	1	1	
A. modicum	1	1	1	1	0	TTTGAACGTTTAGGGCTAGTTTTGAACGATTACTCAACTTTTGAACGATTTGAGA
A. oculi	0	1	1	0	1	
A. palmae	0	1	1	0	1	
M. agalictiae	0	1	1	1	1	
M. arginini	1	1	1	1	0	ATAGCAGAAAGTGCTATAATAGTGTATAATATAATCA
M. arthritidis	0	1	0	0	0	
M. bovis	0	1	1	1	0	
M. callifornicum	F*	0	0	0	F*	TGTCAAGTAGAGGACATAAAGTCCTTATATCAGCT
M. capricolum	0	0	0	0	1	
M. conjunctivae	1	1	1	0	1	CTCAAGTTTTTTGTTATAATTAAACAAGATAAAAAGC
M. crocodyli	0	1	1	1	1	
M. fermentans	1	1	1	1	0	AAAAAATAAAATGTTATAATTTGTTATAAGTTGTTAG
M. gallinarum	1	0	0	0	1	CGACTAAAAAGTGCTAAAAATGTGATATAATTGTGGC
M. gallisepticum	1	1	1	1	0	TATCGGCTTTGTGTTAAAATAGTGTTAACGATTTTGA
M. genitalium	0	0	1	0	1	
M. hominis	1	0	0	0	1	AATCGACAAAGTGATAGTTTTGTGATATAGTTAAGAT
M. hyopneumonia	0	0	0	0	1	
M. hyosynoviae	1	0	0	0	1	TATAGAGTAAATGTAATAAAAATGATATAATTTTGTC
M. imitans	0	1	1	0	1	
M. mobile	F*	1	1	1	F*	TTGTATATTTTAACACACAAATGTTATAATGTAATT
M. mycoides	0	0	0	0	1	
M. penetrans	0	1	1	1	1	
M. pneumonia	0	1	1	1	1	
M. pulmonis	0	1	1	1	1	
M. simbae	F*	0	0	0	F*	TGTTAATAAGCGGACAAATAGTCCTAAATTAATAA
M. synoviae	0	0	0	0	1	
U. canigenitalium	F*	1	0	0	F*	TAGTTATAATATGTCATATTAGTGTCATATATGAATT
U. urealiticum	0	1	1	1	0	

* - Fusion of controller subunit with methylase subunit.
