# Supplementary material for: Binding site of restriction-modification system controller protein in Mollicutes
Source: BMC Microbiol. 2017 Jan 31;17:26. doi: 10.1186/s12866-017-0935-4 (PMC5282649; doi:10.1186/s12866-017-0935-4)
Supplement: Additional file 2: Figure S1. — EMSA with mutations in a single repeat of HsdC binding site. (PDF 143 kb) [file 12866_2017_935_MOESM2_ESM.pdf]

Supplementary figures

A

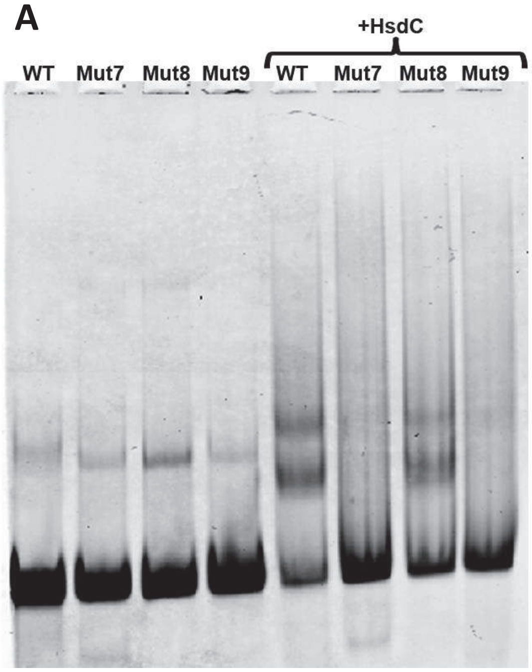

B

HsdC-WT
TTATCGGCTTT**GTGTTA**AAATA**GTGTTA**ACGATTTTGAAG

HsdC-Mut7
TTATCGGCTTT**GacTTA**AAATA**GTGTTA**ACGATTTTGAAG

HsdC-Mut8
TTATCGGCTTT**GaGTTA**AAATA**GTGTTA**ACGATTTTGAAG

HsdC-Mut9
TTATCGGCTTT**GTcTTA**AAATA**GTGTTA**ACGATTTTGAAG

Supplementary figure 1. A – EMSA with mutations in a single repeat of HsdC binding site. Series of four lanes on the left represent negative control (no protein added). HsdC protein was added to the four lanes on the right, marked as **+HsdC**. B – Oligonucleotides used for EMSA. HsdC-binding repeats are underlined; mutations are shown in red and lowercase.
