# Supplementary material for: Binding site of restriction-modification system controller protein in Mollicutes
Source: BMC Microbiol. 2017 Jan 31;17:26. doi: 10.1186/s12866-017-0935-4 (PMC5282649; doi:10.1186/s12866-017-0935-4)
Supplement: Additional file 3: Figure S2. — Binding constant determination for HsdC. (PDF 213 kb) [file 12866_2017_935_MOESM3_ESM.pdf]

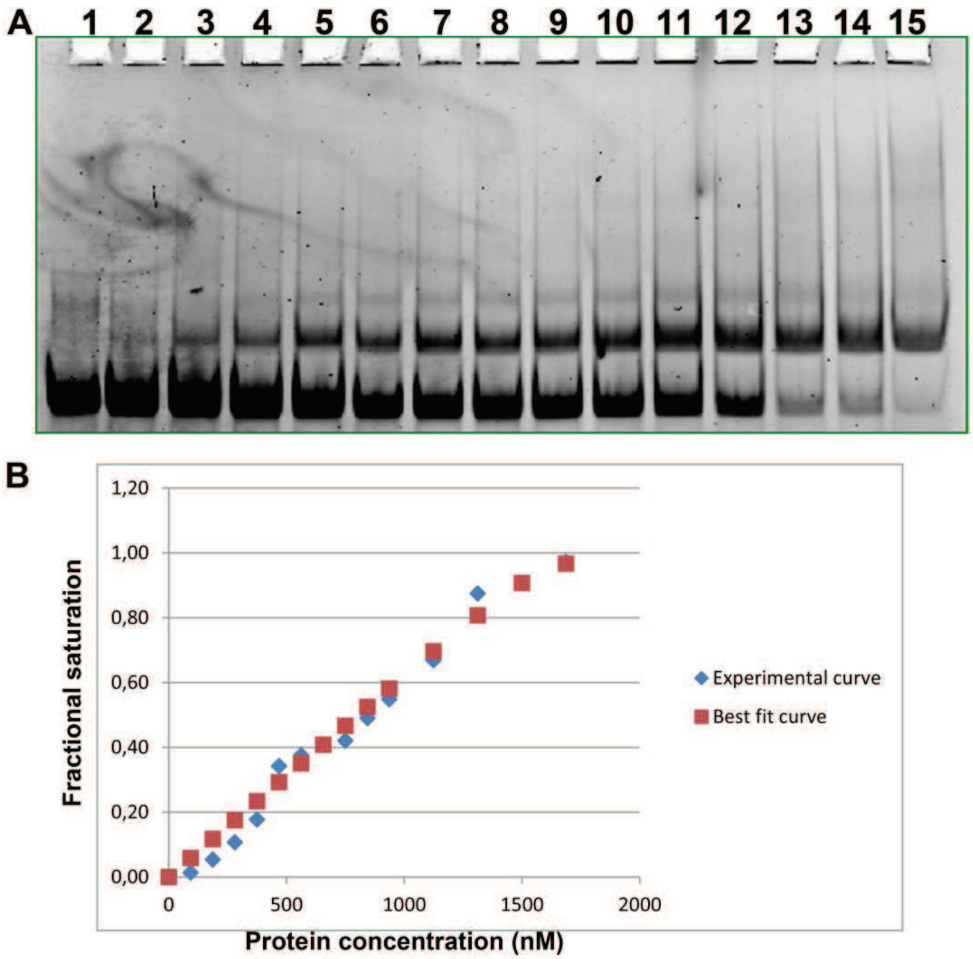

Supplementary figure 2. Binding constant determination for HsdC. A – series of EMSA with different protein dilutions 1 to 15: 0, 93.75, 187.5, 281.25, 375, 468.75, 562.5, 656.25, 750, 843.75, 937.5, 1125, 1312.5, 1500, 1687.5 nM. B – Experimental curve and best fit approximation obtained by least squares method.
