# Supplementary material for: Binding site of restriction-modification system controller protein in Mollicutes
Source: BMC Microbiol. 2017 Jan 31;17:26. doi: 10.1186/s12866-017-0935-4 (PMC5282649; doi:10.1186/s12866-017-0935-4)
Supplement: Additional file 4: Figure S3. — Distribution of promoter powers with strong consensus. (PDF 157 kb) [file 12866_2017_935_MOESM4_ESM.pdf]

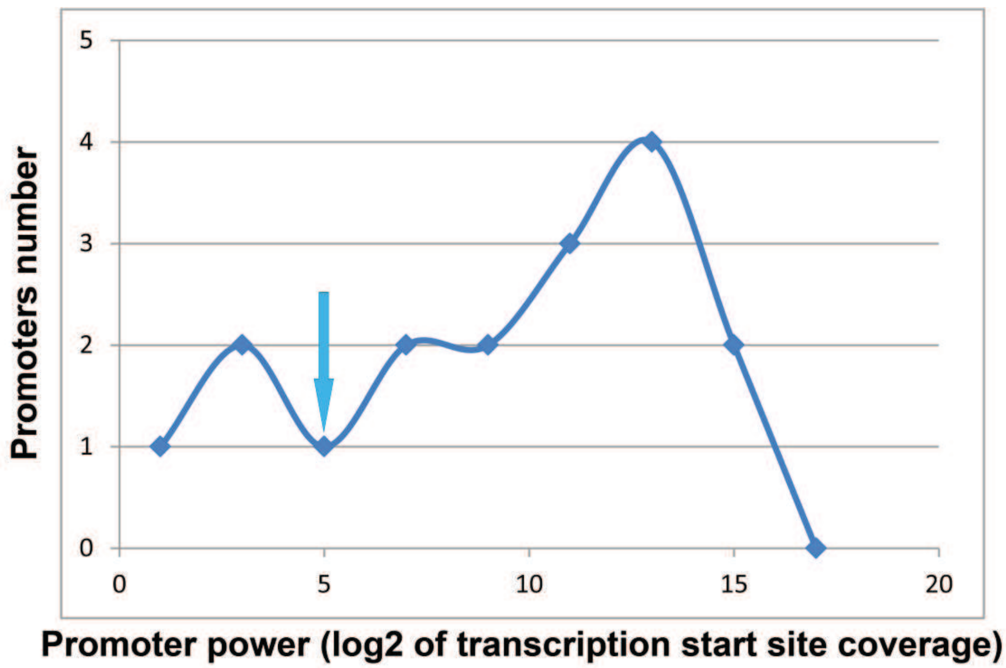

Supplementary figure 3. Distribution of promoter powers measured as base 2 logarithm of transcription start site coverage in 5'-end enriched RNA sequencing (Mazin et al, NAR 2014). The promoters taken for the analysis are full consensus promoters as the promoter of *hsd* operon (TRTGNTAWAATN$_6$R). Arrow indicates measured power of HsdC promoter, which is significantly lower than it would expected. Raw data is shown on Supplementary table 2.
