# Supplementary material for: Binding site of restriction-modification system controller protein in Mollicutes
Source: BMC Microbiol. 2017 Jan 31;17:26. doi: 10.1186/s12866-017-0935-4 (PMC5282649; doi:10.1186/s12866-017-0935-4)
Supplement: Additional file 5: Figure S4. — Genomic rearrangements near hsd (GCW_02350 – GCW_02365) operon insertion. (PDF 176 kb) [file 12866_2017_935_MOESM5_ESM.pdf]

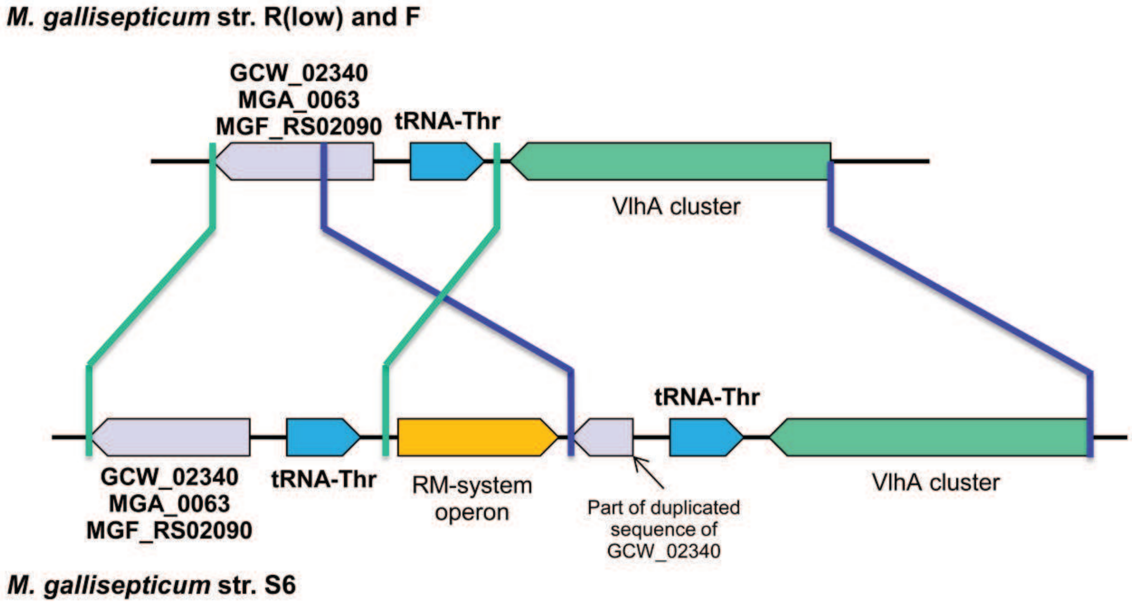

Supplementary figure 4. Genomic rearrangements near *hsd* (GCW_02350- GCW_02365) operon insertion in *M. gallisepticum* S6 strain in comparison to R(low) and F strains. Uptake of *hsd* operon resulted in a large genomic duplication.
