# Supplementary material for: Binding site of restriction-modification system controller protein in Mollicutes
Source: BMC Microbiol. 2017 Jan 31;17:26. doi: 10.1186/s12866-017-0935-4 (PMC5282649; doi:10.1186/s12866-017-0935-4)
Supplement: Additional file 6 — Table S2. Distribution of promoter strengths with strong consensus (TRTGNTAWAATN6R). (PDF 85 kb) [file 12866_2017_935_MOESM6_ESM.pdf]

Supplementary table 2. Distribution of promoter strengths with strong consensus (TRTGNTAWAATN$_6$R). Rep1, Rep2 – biological replicates of 5'-end enriched sequencing. We included promoters of *clpB* and *trnM* (additional targets of HsdC), which have additional substitutions in comparison to consensus.

Promoter	Rep1	Rep2	Gene
TRTGNTAWAAT			
TGTTGTACAAACTGATTTGTTGTGCTATAATCTATAAGGC	32511	26839	23S rRNA
AAGCTAGATCTAAAAAAGAATGTGTTATAATTACAAGGAT	28610	19718	infC operon
ATCATAGATTTCACAATTTTTATGTTATAATTTTATGGGT	13307	18499	trnY operon
TTTAAAAAAACTATTGCTGATATGTTAAAATAGTTAAGGT	9816	8620	rpmB
TTAACTTTGAAATCTAATTGTGTGGTATAATTTTAATGAT	5505	5415	rpsO operon
CTTTAATGAGCTGAATTAATTATGTTATAATTCATATGGC	4270	4471	trnI operon
TAGCCAATAATCAATAATATTATGGTATAATTTTAAAGGC	3429	2748	trnN operon
TTCAATAATTAGAATTCTTATATGTTATAATAAACCTACA	2805	3489	gyrB operon
ATTATTAGAGTGCTAATTATTATGGTATAATATTATTTAG	845	2881	lon
TAAGTAATTTTACTTAACGATATGTTATAATATTTCAGTT	700	1015	rpsP operon
ATCTTTTCAAACGAAAATATTGTGCTATAATTTTAGAGGC	168	388	16S rRNA (gene 1)
GGGTTGGTGCTAGTTAATTTTGTGATAAAATTATACCATC	128	65	dps
ATCTTTACATTTCAAAAATCTGTGCTATAATTATTATAGT	65	99	trnL
TATCGTATTATTATCGGCTTTGTGTTAAAATAGTGTTAAC	36	58	**hsd operon**
ATGATGCGATCGGTGAAGTGTATGATAAAATTGGCCGAGC	12	6	intragenic promoter
CTGAGCCATCAGGAATTCCTTATGCTAAAATCGACTACAA	7	18	intragenic promoter
GTTTAGGTTTAGTTGACTATTATGATAAAATTGTGTAATT	0	2	GCW_03840
AATAGCCTAAGTGCTAATTTTTTGTTATAATAAATCTATT	1373	3248	clpB
CTAATGATTTTACACATTGTTATTATATGATAATAATGTG	472	812	trnM operon
ATTTTTTCAAATTAATTTTTTGTGTTAGAATATTTGAGGT	3894	2308	16S rRNA (gene 2)
